# Severe acute respiratory coronavirus virus 2 (SARS-CoV-2) seroprevalence among healthcare workers in a low prevalence region

**DOI:** 10.1017/ice.2020.1374

**Published:** 2020-12-14

**Authors:** Emily J. Godbout, Rachel Pryor, Mary Harmon, Alison Montpetit, Joan Greer, Lorin M. Bachmann, Michelle Doll, Michael P. Stevens, Gonzalo Bearman

**Affiliations:** 1 Division of Pediatric Infectious Diseases, Children’s Hospital of Richmond at Virginia Commonwealth University, Richmond, Virginia; 2 Epidemiology and Infection Control, Virginia Commonwealth University, Richmond, Virginia; 3 Clinical and Translational Research, Virginia Commonwealth University Health System, Richmond, Virginia; 4 Division of Clinical Pathology, Virginia Commonwealth University, Richmond, Virginia; 5 Division of Infectious Diseases, Virginia Commonwealth University, Richmond, Virginia

*To the Editor*—Healthcare workers (HCWs) continue to work throughout the coronavirus disease 2019 (COVID-19) pandemic despite the potential risk of acquiring COVID-19. Multiple severe acute respiratory coronavirus virus 2 (SARS-CoV-2) seroprevalence studies in HCWs report a seroprevalence range of 0.8% to 31.2%.^1–4^ We performed a convenience serologic survey of HCWs caring for adult and pediatric patients in an academic medical center to estimate the total burden of prior COVID-19 and to describe characteristics associated with seropositive test results. We enrolled participants from inpatient settings and ambulatory clinics to improve generalizability across our healthcare system.

Virginia Commonwealth University Medical Center (VCU) is an 856-bed academic center in Richmond, Virginia. Targeted enrollment was 2,000 participants, and participants were enrolled from July 27 to October 2, 2020. We identified the first confirmed case of COVID-19 at our facility on March 13, 2020. Our facility managed 727 patients with laboratory-confirmed SARS-CoV-2 infection between the first identified case and at the end of the study period. Our facility began universal SARS-CoV-2 screening for all admitted patients by polymerase chain reaction (PCR) testing on April 27, and this continued throughout the study period. Our facility did not implement universal N95 masking for all HCWs, but rather droplet masks and face shields for all direct patient care. Our hospital policy instructs staff to wear N95 masks when caring for patients with COVID-19 if there is concern for aerosolization. SARS-CoV-2 PCR testing is only performed on symptomatic HCWs or as part of contact tracing in outbreak investigations. During the study period, 255 employees in our healthcare system tested positive for SARS-CoV-2 via PCR.

We enrolled HCWs who directly cared for any patient (adult or pediatric), regardless of the patient’s SARS-CoV-2 status. HCWs without direct patient care or those who had previously tested positive for SARS-CoV-2 via PCR were excluded. Participants filled out electronic survey questions about demographic characteristics, role and years of experience, exposure history, and history of symptoms consistent with COVID-19. Participants underwent phlebotomy for serum collection on our clinical research unit. All serum samples were tested at our institution’s laboratory utilizing the Abbott Architect SARS-CoV-2 IgG antibody immunoassay (Abbott Molecular, Des Plaines, IL). We used descriptive statistics to describe participants, stratified by SARS-CoV-2 antibody result. We compared groups using the Fisher exact test or χ2 test for categorical variables, and we used the Student *t* test for continuous variables to identify potential risk factors associated with SARS-CoV-2 seropositivity. We used Research Electronic Data Capture (REDCap) data collection tools hosted at VCU for survey and data collection^8^ and SAS version 9.4 software (SAS Institute, Cary NC) to analyze the data. The VCU Institutional Review Board approved this study.

We enrolled 1,962 participants, including 937 nurses (47.8%), 490 physicians (25.0%), 283 other HCWs (14.3%), 141 advanced practice providers (7.2%), 86 care partners (4.4%), and 25 respiratory therapists (1.3%). Among them, 1,360 (69.3%) self-reported providing direct patient care to a patient with COVID-19. We identified 27 participants (1.4%) with detectable SARS-CoV-2 antibodies. Demographics were similar among those with positive serology and negative serology (Table 1). History of symptoms of fever, cough, or shortness of breath since February 2020 were more prevalent in participants with antibodies detected, (44.4% vs 20.5%; *P* = .002), and those with antibodies detected were more likely to believe that they had previously had COVID-19 (33.3% vs 9.5%; *P* ≤ .001). There was no difference among HCWs who worked on high-risk units, those with the highest number of COVID-19 patient days, versus low-risk units and clinics (2.1% vs 1.1%; *P* = .098) or among HCWs who worked on adult versus pediatric units (1.5% vs 2.2%; *P* = .429).

**Table 1.** Comparison of Characteristics of Enrolled Participants by SARS-CoV-2 Serology Result

Characteristic	SARS-CoV-2 Serology		
	Positive (n=27)	Negative (n=1935)	*P* Value
Mean age, y	37.4 (range, 21–64; SD, 13.0)	37.3 (range, 19–75; SD, 11.4)	.962
Sex, female, no. (%)	19 (70.4)	1,497 (77.4)	.923
Median No. household members, no.	2.59 (range, 1–6; SD, 1.34)	2.74 (range, 0–9; SD, 1.37)	.666
**Age of household members, no. (%)^a^**			
0–5 y	6 (22.2)	392 (20.2)	.799
6–12 y	5 (18.5)	337 (17.4)	.879
13–18 y	2 (7.4)	243 (12.6)	.422
19–65 y	19 (70.4)	1491 (77.0)	.415
>65 y	0 (0)	68 (3.5)	.321
Mean healthcare experience, y	12.0 (range, 1–43; SD, 12.6)	11.1 (range, 0–50; SD, 10.2)	.637
Clinical role, no. (%)			.908
Care partner	2 (7.4)	84 (4.3)	
Nurse	13 (48.1)	924 (47.8)	
Physician	7 (25.9)	483 (25.0)	
Advanced practice provider	2 (7.4)	139 (7.2)	
Respiratory therapist	0 (0)	25 (1.3)	
Other^b,c^	3 (11.1)	280 (14.2)	
Provided direct care to COVID-19 patient, no. (%)	22 (81.5)	1,338 (69.1)	.168
**How many COVID-19 patients cared for, no. (%)**			.355
1–9 patients	14 (51.2)	850 (43.9)	
10–19 patients	2 (7.4)	203 (10.5)	
>20 patients	6 (22.2)	607 (31.4)	
COVID-19 exposure at work, no. (%)	19 (70.4)	1,255 (64.5)	.551
Was appropriate PPE worn during work exposure, no. (%)	10 (76.9)	656 (71.4)	.665
**Reported illness of fever, cough and/or SOB since Feb. 2020, no. (%)**	**12 (44.4)**	**396 (20.5)**	**.020**
Had a negative SARS-CoV-2 PCR test, no. (%)	4 (14.8)	545 (28.1)	.125
**Believe they had a COVID-19 infection, no. (%)**	**9 (33.3)**	**183 (9.5)**	**<.001**
Exposure outside work, no. (%)	3 (11.1)	101 (5.2)	.175
Travel outside US after Jan 2020, no. (%)	1 (3.4)	26 (1.3)	.325
**Maintain social distancing outside of work, no. (%)**			.259
Always	14 (51.2)	700 (36.2)	
Frequently	12 (44.4)	1,205 (62.3)	
Rarely	1 (3.7)	29 (1.5)	
Never	0 (1)	1 (0.1)	

Note. SARS-CoV-2, severe acute respiratory syndrome coronavirus 2; SD, standard deviation; COVID-19, coronavirus disease 2019; PPE, personal protective equipment; SOB, shortness of breath; PCR, polymerase chain reaction.

a This variable has multiple answer options that are not mutually exclusive.

b Other clinical roles included: social worker, dietician, chaplain, transporter, case manager/navigator, radiology technician, clinical technician, emergency medical technician, clinical research role, registration and admitting, lactation consultant, audiologist, child life specialist, clinical pharmacist, other therapists and police/security.

c The other healthcare workers who tested positive by serology included: 1 clinical pharmacist, 1 case manager and 1 therapist (nonrespiratory).

The seroprevalence of SARS-CoV-2 in HCWs at a large, academic medical center in a low-prevalence region was low (1.4%) and comparable to our community prevalence based on a large serologic survey of 4,685 adult Virginians^7^ (1.4% vs 2.4%; *P* = .011). Thus, our current infection prevention strategies are likely effective at preventing patient to HCW transmission. Most serology-positive participants (55.5%) were asymptomatic, suggesting that HCWs may be an important reservoir for HCW-to-HCW transmission in the hospital setting. Serology-positive participants were more likely to believe they had COVID-19 and to have clinical symptoms consistent with COVID-19, yet they infrequently had SARS-CoV-2 PCR testing (14.8%), which highlights the need to continue to address presenteeism in the workplace.

This study has several limitations. It was conducted in a single-center setting, with potential for selection bias and exclusion of HCWs who previously tested positive for SARS-CoV-2 via PCR. Testing was offered on a voluntary basis, and HCWs with a lower or higher risk for infection may have been more likely to volunteer. Exclusion of HCWs with a history of a laboratory-confirmed COVID-19 infection may have led to an underestimation of SARS-CoV-2 seroprevalence. Furthermore, the true prevalence may not be captured with serologic testing due to transient antibody response, which has been documented in HCWs,^9^ and it is a shared feature with circulating seasonal coronaviruses that are associated with the common cold.

The circumstances and unprecedented demands of the pandemic on HCWs is high, and ensuring HCWs are protected from infection is imperative. Our study demonstrates comparable rates of COVID-19 among HCWs and our local community, suggesting that our infection prevention strategies offer protection, including universal droplet mask or face shields, and reservation of N95 masks for patients with COVID-19 with aerosolizing device or procedures. Exposure sources likely expand outside the workplace, and most seropositive HCWs were asymptomatic, potentially serving as reservoirs for transmission in the hospital setting.
